# Individualized procedures for splenic artery dissection during laparoscopic distal pancreatectomy

**DOI:** 10.1186/s12893-020-00694-y

**Published:** 2020-02-13

**Authors:** Yusuke Wada, Takeshi Aoki, Masahiko Murakami, Akira Fujimori, Tomotake Koizumi, Tomokazu Kusano, Kazuhiro Matsuda, Koji Nogaki, Tomoki Hakozaki, Hideki Shibata, Kodai Tomioka

**Affiliations:** Division of Gastroenterological Surgery and General Surgery, Department of Surgery, School of Medicine, Showa University, 1-5-8, Hatanodai, Shinagawa-ku, Tokyo, Japan

**Keywords:** Laparoscopic distal pancreatectomy, Splenic artery, Individual prodedure, Three-dimensional imaging

## Abstract

**Background:**

There are no established standard criteria for choosing the most appropriate procedure of splenic artery dissection during laparoscopic distal pancreatectomy (LDP). The aim of this study was to evaluate the clinical benefits of individualized procedures for splenic artery dissection during LDP based on the variations in arterial structure visualized on preoperative three-dimensional computed tomography (3D-CT).

**Methods:**

Patients who underwent LDP following 3D-CT at a single center were retrospectively evaluated. 3D-CT images were used to construct virtual 3D laparoscopic images for surgical planning. The splenic artery was classified into two major anatomic types: type S that curves and runs suprapancreatic and type D that runs straight and dorsal to the pancreas. Splenic artery dissection was planned according to these two variations, with type S dissected using an suprapancreatic approach and type D using a dorsal approach.

**Results:**

Type-specific dissection was applied for 30 patients: 25 (83%) with type S and 5 (17%) with type D splenic artery anatomies. In 25 (83%) patients, the splenic artery was successfully dissected using the planned surgical procedure, whereas the surgical plan had to be altered in 5 cases (17%) due to difficulty in dissecting the splenic artery.

**Conclusion:**

The individualized procedures for splenic artery dissection according to anatomic variations visualized on 3D-CT images can help improve the success and safety of LDP.

## Background

Laparoscopic distal pancreatectomy (LDP) is widely used as a less invasive alternative to open pancreatic surgery [1–3] with well-documented efficacy and safety [4–9]. Usually, either a ventral or dorsal surgical approach is used for splenic artery dissection during LDP, but there are no established criteria for choosing one over the other. Thus, the approach is generally determined according to the surgeon’s preference, while a more standardized approach may improve surgical success.

Preoperative imaging studies are important for patient-specific surgical planning and simulation, and previous studies have demonstrated the efficacy of preoperative three-dimensional (3D) virtual laparoscopic images [10]. Therefore, we examined individual anatomic variations of the splenic artery using preoperative 3D computed tomography (CT) images and strategically selected two different approaches for splenic artery dissection depending on to the anatomy of the artery. The aim of this study was to evaluate the efficacy and safety of these individualized approaches for splenic artery dissection during LDP.

## Methods

### Patients

Consecutive patients who underwent LDP with presurgical 3D-CT between July 2009 and December 2018 were retrospectively examined. Patients in whom the use of contrast agent was contraindicated were excluded.

### Construction of virtual laparoscopic images

Preoperative CT was performed in all patients to examine individual variations in arterial, venous, and pancreatic anatomies. The preoperative CT imaging protocol included performing CT using a 64-row multi-detector CT scanner (Somatom Definition AS; Siemens) after systemic injection of a nonionic contrast agent (Iomeron (350mgI/135 ml), 1.8 ml/kg; Eisai) at a rate of 4 mL/s with the following imaging parameters: 100 kV, 400 mA, section thickness of 0.75 mm, and collimation of 0.7 mm. The images were then uploaded to Synapse Vincent image processing software (FUJIFILM Medical Co., Tokyo, Japan) to review reconstructed pancreatic structures (i.e., pancreatic parenchyma, splenic vessels, and tumor tissue) and generate virtual laparoscopic 3D geometries that accurately represented the cartography of the pancreas. These images allow surgeons to confirm both the pancreatic parenchymal anatomy and the spatial relationship between vessels and tumors for enhanced safety during the procedure.

### Variations of the splenic artery

In accordance with the virtual laparoscopic images, we classified the splenic artery into two major anatomic types as per a previous study: type S that curves and runs suprapancreatic and type D that runs straight and dorsal to the pancreas [11].

### Selection of the surgical procedure

**Fig. 1:**
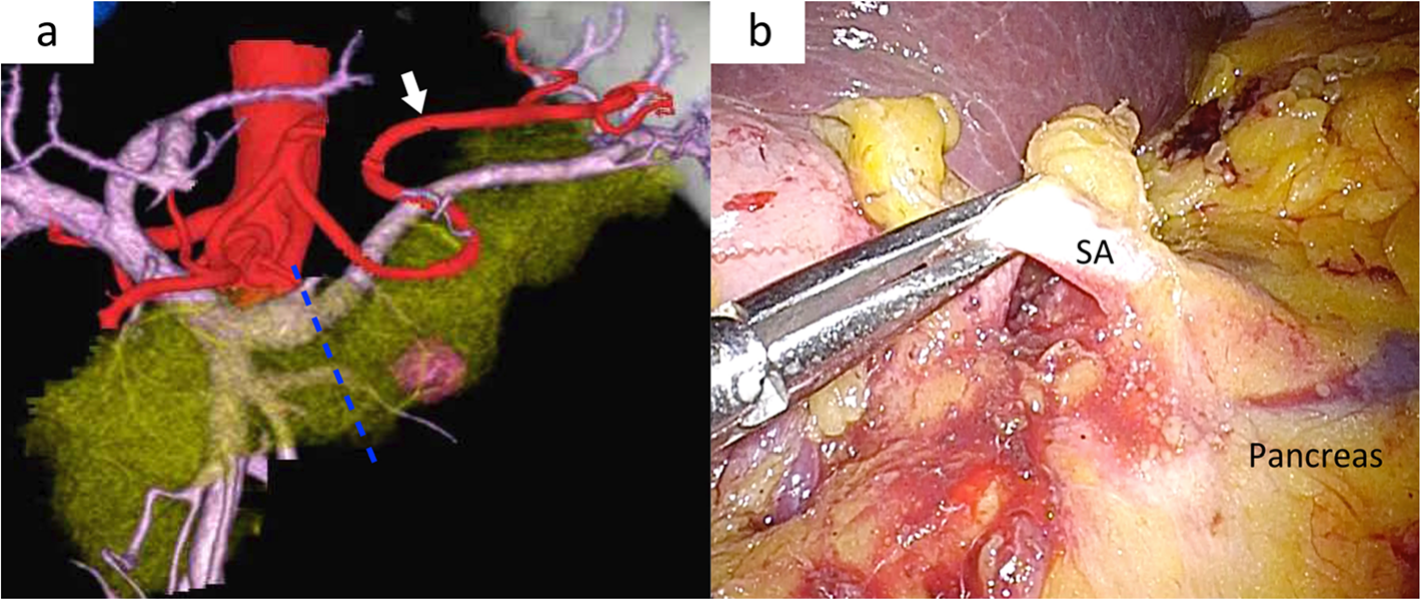
Virtual laparoscopic image and intraoperative findings for type V. **a** Virtual laparoscopic image showing type V. White arrow showing the splenic artery that curves and runs ventral to the pancreas. Blue dotted lines showing the planned dissecting line of pancreatic parenchyma. **b** Intraoperative laparoscopic findings showing the ventral approach. The splenic artery (SA) can be observed ventrally of the pancreas.

**Fig. 2:**
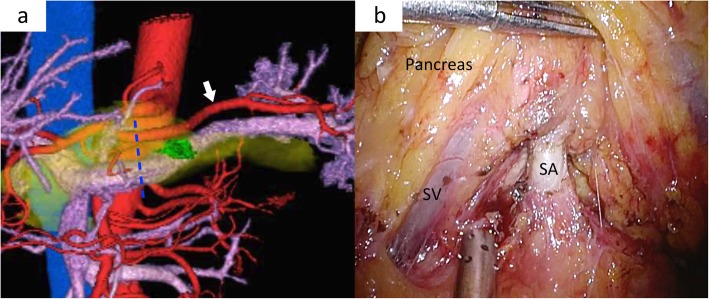
Virtual laparoscopic images and intraoperative findings for type D. **a** Virtual laparoscopic image showing type D. White arrow showing the splenic artery that runs straight and dorsal of the pancreas. Blue dotted lines showing the planned dissecting line of pancreatic parenchyma. **b** Intraoperative laparoscopic findings showing the dorsal approach. The splenic artery (SA) can be observed dorsally of the pancreas and splenic vein (SV).

Initially, the suprapancreatic approach was used for type S (Fig. 1) and the dorsal approach for type D (Fig. 2) in dissecting the splenic artery. However, if the dissection proved difficult, the surgeon modified the procedure accordingly.

### Operative procedure

Under general anesthesia, patients were placed in the reverse Trendelenburg position with hemi-right lateral rotation. The first trocar was placed in the umbilicus to insert a laparoscope (Olympus Medical Systems, Tokyo, Japan) into the peritoneal cavity; intra-abdominal pressure was set at 12 mmHg. Under direct observation, a 12-mm trocar was inserted at the left lateral edge of the rectus abdominis muscle, followed by the insertion of 5-mm trocars in the bilateral subcostal areas and a 5-mm trocar along the left anterior axillary line. The surgeon stood on the left side of the patient, with the first assistant and camera operator standing on the right side.

The left hepatic lobe was kept retracted using a laparoscopic retractor inserted through the left subcostal trocar. A laparoscopic ultrasonic device (HARMONIC ACE+; Ethicon Endosurgery, New Brunswick, NJ, USA) was used for dissection and vessel coagulation. The greater omentum was divided below the gastroepiploic arch to open a window to the omental bursa, followed by the enlargement of the window to expose the pancreas.

For the suprapancreatic approach, the peritoneum was dissected along the superior pancreatic border to expose the splenic artery at the parenchymal dividing line. The inferior border of the pancreas was also dissected toward the splenic lower pole to liberate the pancreatic parenchyma. The pancreatic body was then separated from the retroperitoneum until the splenic vein could be visualized from the dorsal side.

For the dorsal approach, the splenic artery was exposed from the inferior border of the pancreas separating from the splenic vein. The pancreatic parenchyma was then liberated using the same procedure as used for type V cases.

After separation from the pancreas, the splenic artery was ligated using laparoscopic clips (LIGAMAX 5; Ethicon Endosurgery, New Brunswick, NJ, USA) and dissected. Following splenic artery dissection, the splenic flexure of the colon was taken down to achieve complete mobilization of the distal transverse and proximal descending colon from the tail of the pancreas. The gastrocolic ligament was then incised, completely exposing the tail of the pancreas. The gastrosplenic ligament and short gastric vessels were transected up to the superior pole of the spleen. The distal pancreas was then elevated and transected completely from the retroperitoneum.

For parenchymal dissection, laparoscopic ultrasonography was used to identify the location of the tumor and to reconfirm the surgical resection line that was preoperatively planned according to virtual laparoscopic imaging. A linear stapler was inserted through the left lateral 12-mm trocar. After pre-compression, the pancreatic parenchyma including the splenic vein was dissected. After complete dissection of the spleno-retroperitoneum ligament, the specimen was placed into a retrieval bag and removed through the enlarged umbilical trocar site.

### Postoperative evaluations

Postoperative complications were graded according to the Clavien–Dindo classification [12]. Pancreatic fistulas were defined according to the International Study Group on Pancreatic Fistula definition [13].

### Statistical analysis

Continuous variables (such as age) were compared using independent-samples t-tests and categorical variables (such as sex) using chi-square tests. *P* < 0.05 (two-tailed) was considered significant for all tests. All statistical analyses were performed using JMP ver14 (SAS institute Inc., Cary, NC).

## Results

The patient characteristics and the operative outcomes are shown in Table 1. A total of 30 cases were treated using the individualized approaches based on 3D mapping and anatomic classification of the splenic artery. There were 25 (83%) cases with type S and 5 (17%) with type D splenic artery anatomy. Preoperative virtual laparoscopic images clearly revealed splenic artery course in all cases and were helpful in deciding the surgical procedure. The mean total operative time was 189 (range, 85–270) min and intraoperative blood loss was 156 (range, 5–810) mL. None of the patients suffered from grade C postoperative pancreatic fistula. The mean postoperative hospital stay was 16.1 (8–48) days, and there was no perioperative mortality. In 25 (83%) patients, the splenic artery was successfully dissected using the planned surgical procedure, whereas the surgical plan had to be altered in 5 cases (17%) although preoperative virtual laparoscopic images clearly showed the arterial anatomical types (Table 2).

**Table 1:** Patient characteristics and operative outcomes

	Type S (*n* = 25)	Type D (*n* = 5)
Age, average (range)	67.4 (19–86)	58.8 (47–81)
Sex (male), *n* (%)	4 (16%)	2 (40%)
Tumor location, *n* (%)		
Pb	17 (68%)	2 (40%)
Pt	8 (32%)	3 (60%)
Operative time, minute (range)	184 (85–270)	206 (130–270)
Blood loss, ml (range)		
POPF^a^, *n* (%)		
None	18 (72%)	3 (60%)
A	5 (20%)	1 (20%)
B	2 (8%)	1 (20%)
C	0 (0%)	0 (0%)
Complication^b^, *n* (%)		
None	16 (64%)	3 (60%)
I	1 (4%)	1 (20%)
II	4 (16%)	0 (0%)
IIIa	4 (16%)	1 (20%)
>IIIb	0 (0%)	0 (0%)
Hospital stay, day (range)	16.3 (8–48)	15.6 (9–37)

^a^*POPF* Postoperative pancreatic fistula

^b^ Clavien–Dindo classification

**Table 2:** Successful rate of individualized surgical procedure

	Surgical procedure			
	Suprapancreatic	Dorsal	Successful rate	Overall successful rate
Type S	21	4	84%	83%
Type D	1	4	80%	

## Discussion

Preoperative virtual laparoscopic imaging study clearly revealed the splenic artery course in all cases and was helpful in deciding the dissection procedure. Indeed, the planned approach was successful in the majority of cases (87%), and there were no severe perioperative complications.

A previous study has classified the variations in splenic artery course into four types [14]: suprapancreatic (74.1%), enteropancreatic (18.5%), intrapancreatic (4.6%), and retropancreatic (2.8%) courses. In this study, we classified the splenic artery into two major types for subsequent guidance of the dissection procedure. The proportion of type D was nearly equal to that of the suprapancreatic type in the previous study (83% vs. 74%) [14].

A retrospective case series has examined the efficacy of strategic approaches for spleen-preserving LDP [10]; however, the present study is the first to report individualized approaches for splenic artery dissection during LDP based on preoperative virtual laparoscopic images. Since this study does not have a control group, it is difficult to evaluate if this individual approach improves the safety. However we strongly believe that preoperative imaging enables the surgeon to dissect the splenic artery using the shortest course, thereby avoiding unnecessary contact with pancreatic parenchyma and the splenic vessels. Therefore, an individualized approach for each patient may help reduce operative time and decrease intraoperative blood loss, and consequently improves the safety.

In five (17%) cases, intraoperative difficulty was encountered using the planned approach. Because the tumors were located in pancreatic body in all 5 cases, the location of the tumor might have influenced the procedure. Although preoperative virtual laparoscopic images clearly showed the arterial anatomical types, pancreatic dissecting line had to be reconsidered during surgery due to the difficulty in securing the surgical margin, and consequently required alteration in arterial dissecting procedure. Therefore, we suggest that when the tumor is located closer to the portal vein, the surgeon should be prepared for possible deviation from the planned approach.

Several limitations of this study should be acknowledged. First, this was a single-center study; thus, the results may not be applicable to other centers with different levels of expertise in LDP. Second, the retrospective study design and small sample size may have introduced selection and information bias. Finally, the generally positive outcomes may have resulted more from the operator skill than the choice of approach based on virtual 3D-CT laparoscopic imaging. Additional comparative studies are required to establish standard guidelines for splenic artery dissection during LDP.

## Conclusion

Preoperative 3D imaging-based evaluation of splenic artery variations allows for the selection of the most direct surgical approach. Individualized procedures for splenic artery dissection according to preoperative 3D images may improve surgical efficacy and safety.

## Data Availability

The datasets used and/or analyzed during the current study are available from the corresponding author on reasonable request.

## Abbreviations

3D-CT: Three-dimensional computed tomography
LDP: Laparoscopic distal pancreatectomy
POPF: Postoperative pancreatic fistula
